# Supplementary material for: Digital nursing and midwifery leadership: Protocol for a multi-method exploration of policy implementation and impact on practice in the WHO European Region
Source: PLoS One. 2025 Sep 30;20(9):e0332882. doi: 10.1371/journal.pone.0332882 (PMC12483268; doi:10.1371/journal.pone.0332882)
Supplement: Attachment — Submitted filename: response to 2nd review plos one.docx [file pone.0332882.s002.docx]

**Response to 2^nd^ review**

Please find below all revisions made to the manuscript.

We would like to take this opportunity to sincerely thank both reviewers for generously giving their time and expertise to review our paper. Your constructive and insightful feedback has been invaluable and has significantly strengthened the quality and clarity of this protocol.

| Reviewers comments | Action taken in response to reviewers comments |
| --- | --- |
| 1. Does the manuscript provide a valid rationale for the proposed study, with clearly identified and justified research questions?  The research question outlined is expected to address a valid academic problem or topic and contribute to the base of knowledge in the field.  Reviewer #1: Yes  Reviewer #2: Yes | No action required |
| 2. Is the protocol technically sound and planned in a manner that will lead to a meaningful outcome and allow testing the stated hypotheses?  The manuscript should describe the methods in sufficient detail to prevent undisclosed flexibility in the experimental procedure or analysis pipeline, including sufficient outcome-neutral conditions (e.g. necessary controls, absence of floor or ceiling effects) to test the proposed hypotheses and a statistical power analysis where applicable. As there may be aspects of the methodology and analysis which can only be refined once the work is undertaken, authors should outline potential assumptions and explicitly describe what aspects of the proposed analyses, if any, are exploratory.  Reviewer #1: Yes  Reviewer #2: Yes | No action required |
| 3. Is the methodology feasible and described in sufficient detail to allow the work to be replicable?   Descriptions of methods and materials in the protocol should be reported in sufficient detail for another researcher to reproduce all experiments and analyses. The protocol should describe the appropriate controls, sample size calculations, and replication needed to ensure that the data are robust and reproducible.  Reviewer #1: No  Reviewer #2: Yes | We trust that the additional changes to the sampling section address Reviewer 2’s view on methodology. Many thanks |
| 4. Have the authors described where all data underlying the findings will be made available when the study is complete?  The [PLOS Data policy](https://protect.checkpoint.com/v2/r02/___https://na01.safelinks.protection.outlook.com/?url=https%3A%2F%2Ftrack.editorialmanager.com%2FCL0%2Fhttps%3A%252F%252Fjournals.plos.org%252Fplosone%252Fs%252Fmaterials-and-software-sharing%2F1%2F010f019902e21767-bd3a0675-98aa-4d31-9796-fdc96ed189e5-000000%2F4t2AbALvOTcqBtNs8g13HqmJ-Aytr1WZEV4InGM7T40%3D226&data=05%7C02%7C%7C3593568e54a7442a120208dde9252a7e%7C84df9e7fe9f640afb435aaaaaaaaaaaa%7C1%7C0%7C638923068046592799%7CUnknown%7CTWFpbGZsb3d8eyJFbXB0eU1hcGkiOnRydWUsIlYiOiIwLjAuMDAwMCIsIlAiOiJXaW4zMiIsIkFOIjoiTWFpbCIsIldUIjoyfQ%3D%3D%7C0%7C%7C%7C&sdata=Hi4%2Fzf3SSfUFrMUuq5V9kqp2pWY5GjOqqNzB3ZPzt6A%3D&reserved=0___.YzJlOmFuZ2xpYXJ1c2tpbnVuaXZlcnNpdHk6YzpvOmI5OTFlZWVkMmI5YjI0YTk0M2I1Y2I5MWQxZWNlNDQ3Ojc6OWVlNzowZGEwNjhlZTU2N2NhNzU5ZDNlYjNjYzk2Y2E1MjY0ZmY2ZTg1YzAwZDUxYjBiMjYxMjhiYmI4MmMyMGUyYTc2OnA6VDpG) requires authors to make all data underlying the findings described in their manuscript fully available without restriction, with rare exception, at the time of publication. The data should be provided as part of the manuscript or its supporting information, or deposited to a public repository. For example, in addition to summary statistics, the data points behind means, medians and variance measures should be available. If there are restrictions on publicly sharing data—e.g. participant privacy or use of data from a third party—those must be specified.  Reviewer #1: Yes  Reviewer #2: Yes | No action required |
| 5. Is the manuscript presented in an intelligible fashion and written in standard English?  PLOS ONE does not copyedit accepted manuscripts, so the language in submitted articles must be clear, correct, and unambiguous. Any typographical or grammatical errors should be corrected at revision, so please note any specific errors here.  Reviewer #1: Yes  Reviewer #2: Yes | No action required |
| 6. Review Comments to the Author  Reviewer #1: The sampling process needs to be elaborated. Selection criteria for Phase I study is clear but it should also include how the participants will be selected for phase II. How will the researcher select the minimum of 25 participants from 53 WHO regions? What are the criteria for selection? Is there any incentive for the participants? If yes, please mention. Please in include the guideline for the interview, FGD.  Reviewer #2: I have reviewed the authors' responses to the reviewers' comments and the accompanying revisions to the manuscript. I find their responses to be thorough and the revisions have successfully addressed all concerns raised. All the best. | Thank you for this observation. We have revised the sampling section on page 4 to further strengthen and clarify our approach.  With regards to the focus group discussion/interview guide which will be used in Phase 2. This can not be included as it will be developed in light of the responses obtained in phase 1 of the study. This is stipulated on page 6, under the section heading ‘Phase 2 Online focus group. When the study is completed/reported access to the discussion/interview guide used for the study will be provided.  Many thanks. No further action required |
